# Medical mycology and fungal immunology: new research perspectives addressing a major world health challenge

**DOI:** 10.1098/rstb.2015.0462

**Published:** 2016-12-05

**Authors:** Neil A. R. Gow, Mihai G. Netea

**Affiliations:** 1 Aberdeen Fungal Group, Institute of Medical Sciences, University of Aberdeen, Aberdeen AB25 2ZD, UK; 2 Department of Internal Medicine, Radboud University Nijmegen Medical Centre and Radboud Center for Infectious Diseases (RCI), Nijmegen, 6500HB, The Netherlands

**Keywords:** fungal cell wall, fungal infection, genetic susceptibility, immune recognition, microbiome

## Abstract

Fungi cause more than a billion skin infections, more than 100 million mucosal infections, 10 million serious allergies and more than a million deaths each year. Global mortality owing to fungal infections is greater than for malaria and breast cancer and is equivalent to that owing to tuberculosis (TB) and HIV. These statistics evidence fungal infections as a major threat to human health and a major burden to healthcare budgets worldwide. Those patients who are at greatest risk of life-threatening fungal infections include those who have weakened immunity or have suffered trauma or other predisposing infections such as HIV. To address these global threats to human health, more research is urgently needed to understand the immunopathology of fungal disease and human disease susceptibility in order to augment the advances being made in fungal diagnostics and drug development. Here, we highlight some recent advances in basic research in medical mycology and fungal immunology that are beginning to inform clinical decisions and options for personalized medicine, vaccine development and adjunct immunotherapies.

This article is part of the themed issue ‘Tackling emerging fungal threats to animal health, food security and ecosystem resilience’.

## 1. Introduction

Fungi represent a major threat to human health accounting collectively for more than a billion skin infections, more than 100 million mucosal infections, 10 million serious allergies and more than a million deaths each year. Global mortality owing to fungal infections is greater than for malaria and breast cancer and is equivalent to that for tuberculosis (TB) and HIV [1]. Fungal infections induce a complex set of disease states in which pathology can be the result of fungal virulence factors that cause tissue destruction or, alternatively, can result from inflammation caused by the presence of the fungus [2]. Consequently, it is important to understand the immunopathology of fungal infections in order to be able to consider the opportunities for augmentative immunomodulatory treatments. Few fungi are primary pathogens of healthy humans and most life-threatening fungal infections occur in the immunocompromised patients with trauma, HIV infection, immunosuppression and neutropenia and where the normally protective bacterial microflora is disrupted [1]. To understand the balance between immune surveillance, disease progression, host invasion and pathology, it is therefore important to be able to define the nature of the protective immune response to fungal invaders and other factors that predispose us to infection.

## 2. Induction and suppression of immunopathology

**Figure 1.**
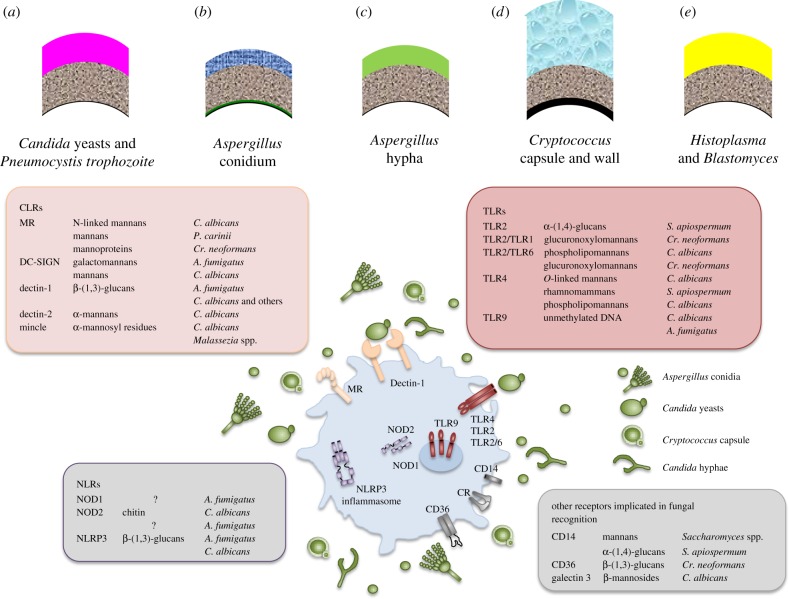
Immune recognition of fungi and their cell walls. (*a–e*) The cell wall structure of a range of fungal pathogens. The conserved inner wall (grey) is composed mainly of β-1,3-glucan and chitin (lacking in the *Pneumocystis* wall). The outer wall is predominantly of mannan (*Candida*, pink); hydrophobins, melanin and α-glucan (*Aspergillus* conidium, dark blue) and galactomannan and galactosaminoglycan (*Aspergillus* hypha, green); capsular glucuronoxylomannan, galactoxylomannan (*Cryptococcus*, light blue) or α-glucan (*Histoplasma* and *Blastomyces*, yellow). PAMP–PRR interactions for fungal cell recognition are shown above. Organism names are as in the text, *S. apiospermum* = *Scedosporium apiospermum*. The figure was provided by Dr Jeanette Wagener.

More than a decade of fungal immunology research has focused on defining the molecular interactions between pathogen associated molecular patterns (PAMPs), which are dominated by component polysaccharides of the fungal cell wall, and their cognate pattern recognition receptors (PRRs) from the toll-like receptor (TLR), C-type lectin (CTL) and nod-like receptor (NLR) families [3–5] (figure 1). Recognition events lead to engulfment of fungal cells, cell signalling, the release of cytokines and other molecules that recruit phagocytes and antigen-presenting cells to the sites of infection, leading to the activation of naive T cells and the induction of antibody production by B cells. Macrophages and neutrophils provide first-line defences killing fungal invaders by attacking fungal cells with variety of enzymes and toxic oxidative and nitrosative compounds. Dendritic cells direct the maturation of naive CD4^+^ T helper cells (T_H_) and regulatory T cell (T_Reg_) populations, leading to both protective and sometimes pathological inflammatory reactions to the presence of a fungus [2]. An important dynamic in fungal immunology is that the pathology caused by a fungal invader can be mediated either by the destructive forces imparted by virulence factors or by the over-activation of the inflammatory response causing collateral damage to host tissue. The recently described candidalysin product of a peptide derived from proteolysis of the Eec1 protein is an example of a fungal virulence attribute that inflicts damage on the host [6]. The polysaccharide β-1,3 glucan, a signature molecule in the cell walls of all fungal pathogens, is a strong activator of inflammation via activation of T_H_17 immune responses and of the NLRP3 inflammasome. These responses are required for immune protection, but can also lead to pathological tissue damage if not subject to attenuation and immunomodulatory regulation [7].

**Figure 2.**
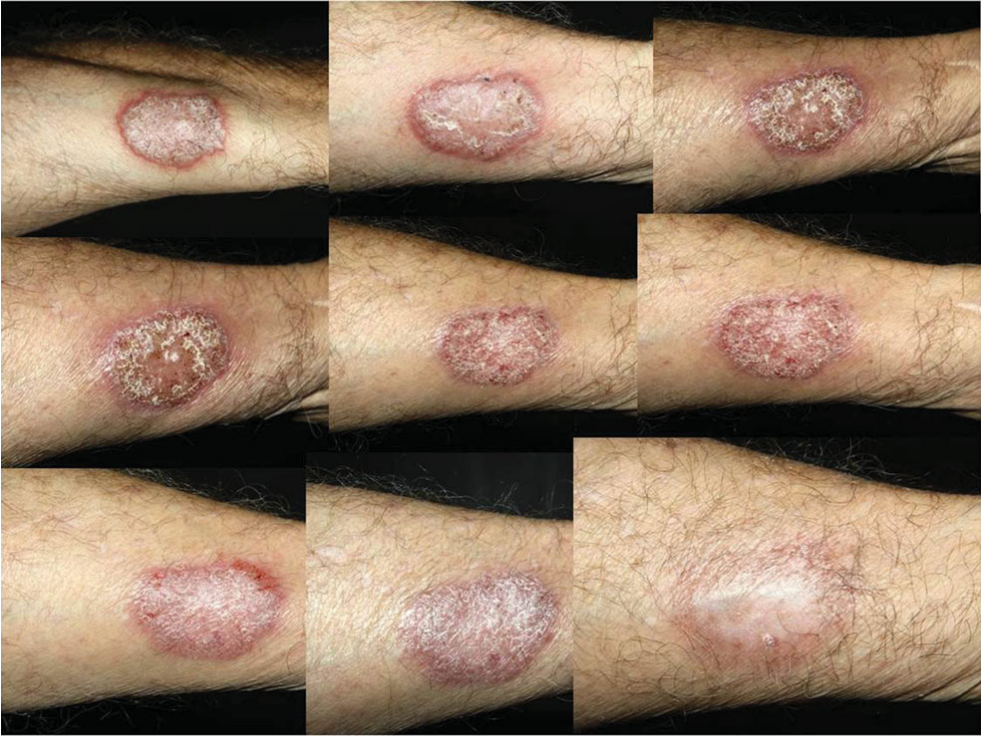
Treatment of chromoblastomycosis from time 0 to 20 months' application of topical imiquimod 5% plus itraconazole 200 mg day^−1^ [9]. With thanks to Paulo R. Criado and Walter B. Júnior and G. de Sousa.

Recent work exemplifies the principle that understanding the nature of the recognition mechanism and immune response can present novel therapeutic options. For example, Brown and co-workers showed that the normal immune response to *Fonsecaea pedrosoi* was inadequate to generate a protective inflammatory response [8]. This fungus is an agent of chromoblastomycosis—a chronic skin infection that is normally highly recalcitrant to treatment with antifungal antibiotics and often requires surgical debridement to effect adequate treatment (figure 2). In a pre-clinical mouse model of *F. pedrosoi* infection, it was shown that intravenous or intraperitoneal injection of bacterial lipopolysaccharide (LPS) augmented the primary recognition of the fungus mediated by the mincle CTL, leading to complete elimination of the fungus [8]. A recent clinical trial has shown that topical administration of the TLR7 agonist Imiquimod, with and without concurrent oral antifungals, was highly active in promoting the elimination of *F. pedrosoi* from skin lesions [9]. It is therefore important to understand the virulence properties and immune recognition of the major fungal pathogens in order to inform augmentative immunotherapy options. At present, our understanding of these areas is dominated by investigations of model pathogens such as *Candida albicans*, and we are not yet able extrapolate this knowledge to be predict how even related *Candida* species induce pathology.

Studies of fungal immune recognition emphasize the importance of several classes of cell wall polysaccharides [3–5]. The outer walls of fungi are chemically diverse and contain a variety of polymers that are either mildly proinflammatory or more or less immunologically inert, providing a mask over the inner cell wall that is normally dominated by the highly proinflammatory β-1,3-glucan layer that is recognized by dectin-1 [10]. Damage to the outer mannan layer of the *Candida* cell wall unmasks β-1,3-glucan, which also occurs naturally when the cell wall is attacked by the lytic enzymes of phagocytes or exposure to antifungal drugs such as echinocandins that damage β-1,3-glucan and hence compromise cell wall integrity [11]. A range of mannosylation defective mutants of *C. albicans,* including *och1*, *pmr1* and *mnn1*, have been shown to have increased exposure and immune recognition of components of the inner cell wall [12,13]. Fungal mannans are mildly proinflammatory and are the natural ligands for a wide range of PRRs, including TRL2, TLR4, mannose receptor, mincle, dectin-2, DC-SIGN and galectin 3 [4] (figure 1). However, fungal β-1,3-glucan is the most immunologically active fungal PAMP, and the full immune response to a fungus does not occur until β-1,3-glucan in the inner cell wall is exposed. Cooperative, simultaneous recognition of multiple cell wall components via receptor complexes results in amplification of the recognition response [14].

A second signature polysaccharide of the inner fungal cell wall is chitin. This is present in variable quantities in different fungi and can be more or less deacetylated to chitosan. Chitin of *C. albicans* and other fungi and invertebrates induced particle size-dependent immune responses from myeloid cells. Larger particles induced TNF, IL-6 and other proinflammatory cytokines, whereas smaller-sized particles induced the anti-inflammatory cytokine IL-10 via a novel receptor signalling pathway involving the mannose receptor, NOD2 and TLR9 [15]. Fungal chitin also induced eosinophilia that may be linked to asthma with fungal sensitization. Administration of highly purified fungal chitin into the peritoneum of mice inhibited the recruitment of inflammatory cells associated with co-administration of LPS [15]. Chitin particles also have been shown to induce IL-10 in the colon and offset the pathology associated with inflammatory gut disorders [16]. Moreover, echinocandin-treated cells of *Candida* and *Aspergillus* upregulate chitin production in their walls to offset damage inflicted on cell wall β-1,3-glucan [17,18]. Such chitin-rich cells may be less inflammatory *in vivo*, thus limiting the immune response required for their elimination by the immune system [17].

Therefore, the therapeutic administration of Imiquimod and chitin provides contrasting examples where specific pathologies associated with different disease types can be treated by either enhancing or suppressing inflammation.

## 3. Genetic susceptibility—the impact of genomics on medical mycology

Not all patients in high-risk groups (see above) develop invasive disease, and these risk factors do not fully explain the susceptibility to candidiasis. Thus, the genetic make-up of the host has also been suggested to play an important role in the susceptibility to infection. In rare cases, single-gene defects in patients leading to primary immunodeficiencies result in significantly enhanced susceptibility to fungal infections. Despite the rarity of such cases, much has been learned about the pathophysiology of fungal infections from understanding the genetic and immunological basis of the defects in these patients.

## 4. Primary immunodeficiency syndromes

Several immunodeficiencies are characterized by a strongly increased susceptibility to fungal infections, among which chronic mucocutaneous candidiasis (CMC), hyper-IgE syndrome (HIES) and chronic granulomatous disease (CGD) are important examples. CMC is a heterogeneous group of clinical syndromes characterized by chronic or recurrent infections of the skin, nails and mucous membranes caused by *Candida* spp., but also by other fungi. Recently, mutations responsible for the impaired immune response have been identified in several of the primary immunodeficiencies associated with CMC. Autoimmune polyendocrinopathy-candidiasis-ectodermal dystrophy (APECED) is an autosomal recessive disorder caused by mutations in the autoimmune regulator (AIRE) gene and that is characterized by CMC, hypoparathyroidism and Addison's disease [19,20]. It is believed that in patients with APECED, T-lymphocyte immunological surveillance fails owing to neutralizing autoantibodies against IFNγ and IL-17, leading to chronic *Candida* infection [21,22]. Autosomal-dominant chronic mucocutaneous candidiasis (AD-CMC) is another CMC syndrome in which mutations in the coiled-coil domain of signal transducer and activator of transcription 1 (STAT1) have been identified as the underlying cause [23,24]. Functional studies in these patients showed defective T-lymphocyte immune responses, such as decreased production of IFNγ, IL-17 and IL-22, important components of antifungal host defence [23]. In addition, defects in IL-17F or IL-17R have also been reported as a rare cause of CMC [25].

HIES is an autosomal-dominant immunodeficiency characterized by high serum IgE, eczema, recurrent *Staphylococcal* skin abscesses, bone and connective tissue abnormalities and recurrent skin and pulmonary infections, especially with *Staphylococcus aureus* and *Candida* species [26]. There are multiple genetic causes of HIES, with mutations in STAT3 being the most common [27,28]. In addition, defects in the dedicator of cytokinesis 8 (DOCK8) [29] and Tyk2 [30] have also been suggested to cause HIES. STAT3 is involved in the signal transduction pathway required for the expression of many cytokine receptors, and patients bearing *STAT3* mutations have almost no T_H_17 lymphocytes and fail to produce IL-17 [16–18]. Based on *in vitro* murine and human studies that showed that IFNγ inhibits IgE production, recombinant IFNγ has been administered to patients with HIES, resulting in improved immunological responses [31–33]. These observations warrant future trials to evaluate the clinical response of HIES patients to recombinant IFNγ supplementation.

CGD is another condition characterized by marked increased susceptibility to invasive bacterial and fungal infections. The fungal infections are mainly represented by *Aspergillus*, with the interesting observation of a high proportion owing to *A. nidulans* rather than to *A. fumigatus* that normally accounts for the great majority of *Aspergillus*-associated human infections [28]*.* CGD is caused by mutations in one of the proteins of the nicotinamide adenine dinucleotide phosphate oxidase complex. Mutations in all five subunits of this complex have been described (gp91phox, p47phox, p22phox, p67phox and p40phox), which result in the loss of function of the complex and defective production of reactive oxygen species. Up to 70% of cases are X-linked, with the remaining being autosomal [34,35]. Management of CGD typically includes lifelong antifungal prophylaxis [36], while patients can also benefit from administration of recombinant IFNγ especially in a prophylactic setting [37]. In addition, hematopoietic stem cell transplantation may offer a longer-term solution for CGD patients [38], whereas gene therapy using retroviral vectors has also been proposed as a future intervention [39]. More research is required to evaluate the long-term safety and effectiveness of gene therapy.

In addition to these classical immunodeficiencies, new studies have described novel forms of immunodeficiency based on defects in specific receptors or their associated intracellular pathways. Among these, one of the most severe is the defect in CARD9—the adaptor molecule crucial for inducing the intracellular signalling of C-type lectin receptors. Defects in CARD9 are associated with significantly increased susceptibility to invasive fungal infections, which is mediated through multiple immunological defects [40,41]. Complete functional defects in one of the important C-type lectin receptors, dectin-1, have also been described to be associated with mucosal *Candida* infections [42,43], but the relatively common prevalence of this mutation and the mild clinical presentation suggest this should be regarded as a mutation associated with an increased susceptibility risk, rather than as a classical primary immunodeficiency.

## 5. Common genetic variation and susceptibility to fungal infections

In addition to primary immunodeficiencies, common variants in (especially) genes coding for proteins involved in the immune system have also been shown to govern relative susceptibility to infection (table 1). These mutations are most often loss-of-function recessive polymorphisms. Several epidemiological investigations have assessed the role of TLR polymorphisms for the susceptibility to disseminated candidiasis. The Asp299Gly TLR4 polymorphism has been proposed to act as a susceptibility trait for systemic candidiasis [56] and the Asp753Gln TLR2 polymorphism resulted in an altered cytokine profile in patients with *Candida* sepsis [57]. However, the hypothesis that these polymorphisms are involved in susceptibility to candidemia could not be confirmed in a much larger cohort of individuals, including patients and matched controls [45]. Similarly, no role of TLR4 polymorphisms in vaginal colonization with *Candida* spp. has been observed [58].

**Table 1.** Common genetic variants associated with increased susceptibility to *Candida* infections. MBL, mannose binding lectin.

affected gene	polymorphism	type of infection	immune defects	references
dectin-1	Y238X	recurrent vulvovaginal infections and oral/gastrointestinal colonization	lack of β-glucan recognition, lower production of TNFα, IL-6 and IL-17	[23]
	I223S	oropharyngeal candidiasis	reduced zymosan-binding capacity and IFN-γ production	[44]
TLR1	R80T N248S S602I	candidemia	impaired production of cytokines	[45]
TLR3	L412F	CMC	defective TLR3 signalling	[46]
IL-12B	−2724INS/DEL	persistent candidemia	lower production of IFN-γ	[47]
IL-10	−1082A/G	persistent candidemia	higher production of IL-10	[47]
MBL2	codon 54 and 57	candidemia, abdominal *Candida* infection, recurrent vulvovaginal candidiasis	lower MBL serum levels	[48–50]
IL-4	−589T/C	recurrent vulvovaginal candidiasis	increased levels of vaginal IL-4 and reduced levels of nitric oxide and MBL	[51]
	−1098T/G −589C/T −33C/T	chronic disseminated candidiasis	unknown	[52]
NLRP3	length polymorphism	recurrent vulvovaginal candidiasis	impaired production of IL-1β	[53]
DEFB1	−44C/G	*C. albicans* carriage	unknown	[54]
CD58. TAGAP	various SNPs in the locus	candidemia	decreased phagocytosis and cytokine production	[55]

Studies dedicated to identifying common genetic variants that predispose to bloodstream *Candida* infections have revealed a significant role for non-synonymous polymorphisms in TLR1 [45]. These TLR1 polymorphisms result in loss-of-function of the receptor, defective pattern recognition of the pathogens, and decreased cytokine production. Another potential mechanism through which TLR1 could exert its effect was discovered through the recent finding that β-defensin-3 activates immune cells through TLR1/TLR2, with an important lytic activity against *C. albicans* [59,60]. This is supported by the observation that polymorphisms in β-defensin-1 are associated with recurrent vulvovaginal candidiasis (RVVC) [54]. In the same cohort of patients with candidemia, persistence of fungemia was shown to be associated with promoter polymorphisms in the cytokine genes IL-12B and IL-10 [47]. These polymorphisms affect cytokine transcription and thereby influence the IL-10 and IL-12 production capacity of innate immune cells [61–65]. The persistence of infection was demonstrated to correlate with decreased IL-12 and increased IL-10 production induced by the presence of *Candida*, which may result in inhibition of the T_H_1 response that is known to be crucial for anti-*Candida* systemic immunity [66,67]. In agreement with this, a decreased production of T_H_2 cytokines such as IL-4 owing to genetic variation in the IL4 gene also leads to protective effects [68,69].

Multiple studies have been dedicated to investigate the role of mannose binding lectin (MBL) deficiency in infections with *Candida* spp., because it was demonstrated that MBL could bind and opsonize fungi to facilitate complement activation and phagocytosis [69,70]. Indeed, genetic associations of MBL deficiency with infection risk have been observed in cohorts of patients with candidemia, abdominal infections and RVVC [48–50,68].

*Candida* spp. also cause frequent mucosal infections that can be an important cause of morbidity [2]. Oropharyngeal candidiasis (OPC), a mucosal colonization of the mouth and upper digestive tract, is frequently observed in patients that are infected with HIV. About 50–95% of patients suffer this type of candidiasis at least once during their progression to AIDS [71–73]. A recent study assessed the potential role of genetic variants of pattern recognition receptors in susceptibility to OPC in West African HIV patients, and revealed an I223S genetic variant of dectin-1 that was specific for African populations [44].

The genetic studies on *Candida* infections described above are variable in terms of size of patient cohorts and statistical power. While some of the studies had relatively large cohorts, with appropriate statistical analysis, others were based on relatively small numbers of patients and this limited the robustness of the conclusions. Although some of the reported genetic associations were supported by functional studies that provided mechanistic explanations for the increased susceptibility to infection, the common lack of genetic validation of these genetic associations underlines the importance of future studies with appropriate statistical power using independent cohorts of patients.

## 6. Genomic approaches in fungal infections

In addition to the classical candidate-based approaches for investigating susceptibility to fungal infections, recent methodological advances have permitted the initiation of discovery-based genomic approaches to identify novel genetic variants that impact on fungal disease. Recently, the first genome-wide association study (GWAS) on a fungal infection was published [55], in which the authors analysed and compared 118 989 single-nucleotide polymorphisms (SNPs) in patients with candidemia and a large cohort of healthy volunteers. A significant association between candidemia and SNPs in cluster of differentiation (CD) 58 (odds ratio, OR = 4.68), late cornified envelope 4A (LCE4A; OR = 4.25) and T cell activation RHO-GTPase-activating protein (TAGAP; OR = 2.96) loci was identified. The combination of two or more risk alleles in these two genes resulted in an almost 20-fold increase in the risk for candidemia [55]. CD58, and adhesion molecule on antigen-presenting cells, appeared to be involved in the inhibition of *Candida* germination, whereas TAGAP was needed for optimal *Candida*-induced cytokine production [55]. More studies are needed to validate these findings, to expand the depth of these genomic approaches to more genetic variants, and to perform GWASs also in other fungal infections. Such studies may pave the way for future clinical decisions based on personalized SNP profiling.

## 7. Understanding the impact of microbiome on the pathophysiology of fungal infections

**Figure 3.**
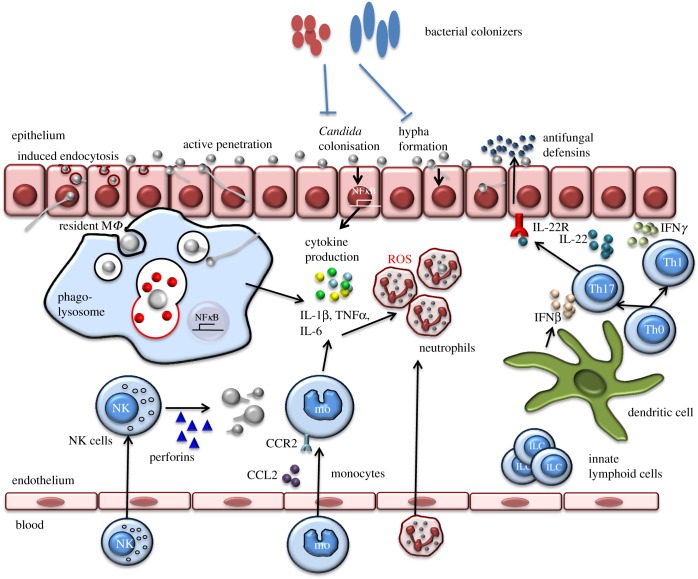
Microbiomes and immunology. Direct and indirect influences of the microbiome on the physiology of fungal pathogen growth and the innate immune response as described in the text. In short, colonization with *Candida* does not induce the production of cytokines. Upon invasion, *Candida* activates tissue macrophages to induce production of chemokines and cytokines. This, in turn, will recruit and activate other immune cells from the bloodstream, such as monocytes or neutrophils that will ingest and kill the pathogens. Activations of dendritic cells will also lead to antigen presentation and activation of T-helper responses that, in turn, will aid pathogen elimination. CCR2, chemokine receptor type 2; CCL2, chemokine (C–C motif) ligand 2; NK = natural killer cell; ROS, reactive oxygen species.

Recent years have witnessed a revolution in our understanding of how the microbiome composition impacts the health status of the host [74]. The microbiome composition is strongly influenced by diet, and this can influence colonization of mucosae with fungi (figure 3). For example, a recent study in mice showed that dietary coconut oil reduced *C. albicans* colonization of the gastrointestinal tract [75]. *Candida* colonization is also influenced by quantitative and qualitative aspects of the microbiome, such as *Lactobacilli* that inhibit fungal adhesion and growth by producing H_2_O_2_ and bacteriocin-like compounds [76]*.* Another example through which microbiome products influence fungal colonization is that of the production of short-chain fatty acids by *Lactobacilli* that can also inhibit fungal growth [77]. Some bacteria such as *Pseudomonas aeruginosa* and *Enterococcus faecalis* can inhibit *C. albicans* hypha formation and thereby have the potential to influence tissue infiltration [78]*.* Supporting this, in a *Caenorhabditis elegans* infection model, *E. faecalis* in the gut inhibited *C. albicans* hyphal morphogenesis [79]. In contrast, *C. albicans* can co-aggregate with *Streptococci*, which may facilitate the colonization of oral surfaces by the yeast [80].

The microbiome composition not only influences the extent of *Candida* colonization on human tissues, but it can also influence the immune response to *Candida* species [81–83] (figure 1)*.* Mice treated with the short-chain fatty acid propionate, a product of the metabolism of dietary fermentable fibres by many gut microorganisms, have numerous immune alterations, including enhanced generation of macrophage and dendritic cell precursors, increased number of dendritic cells in the lungs and reduced T_H_2 effector function [84]. *Lactobacilli* in the gut use tryptophan as their energy source and produce indole-3-aldehyde in the process. Indole-3-aldehyde stimulates the aryl hydrocarbon receptor, which induces IL-22 production in NKprotein46^+^NK1.1^low^ cells, which are, in turn, protective against *Candida* colonization at mucosal surfaces [85]. Thus, bacterial components of the microbiome can influence immune responses in the gut and in the lung.

The microbiome composition in humans has also been demonstrated to influence the immune response against *C. albicans.* In patients with hyper-IgE syndrome and CMC, there is an increase in skin colonization with Gram-negative bacteria (e.g. *Acinetobacter*), whereas some of the regular skin microbiome genera (e.g. *Corynebacteria*) have a much lower prevalence than in controls. This pathological colonization with Gram-negative bacteria can, in turn, suppress *S. aureus*-and *C. albicans*-induced cytokine production [86]*.* The vaginal microbiome of patients with vulvovaginal candidiasis is highly variable, and could not be described by any single profile [87]*.* In terms of abundance, fungi are relatively rare on human skin [88], and the fungal microbiome has yet to be researched in detail.

When the normal immune balance is disturbed, e.g. in immunocompromised hosts, populations of resident skin fungi can expand [89]. Indeed, antibiotic treatment in mice causes an altered gut microbiome that coincides with outgrowth of commensal *Candida* species in the gut [90]*.* In addition, genetic factors contribute to mycobiome composition. Fr example, in dectin-1 deficient mice, there is an altered gut mycobiome, which subsequently increases the susceptibility to experimental colitis [91].

Oral mycobiome analysis revealed that *C. albicans* was the most common fungal microorganism in healthy controls and HIV-infected participants. *Candida* colonization is negatively correlated with the abundance of *Pichia farinosa*—a near relative of *Candida boidinii*. *Pichia*-conditioned medium is a medium in which the yeast *Pichia* has been grown and then removed by filtration. Interestingly, *Pichia-*conditioned medium inhibits *Candida* growth and biofilms, and in a murine oral candidiasis model, *Pichia-*conditioned medium lowered the infection score, fungal burden and tongue epithelial damage [92]*.* This study provides proof of principle that investigating the relationship between the bacteriome and mycobiome, or between various components of the mycobiome, can both provide new insights into the pathology of a disease and can lead to novel antifungal approaches [92].

## 8. Conclusion and future prospects

Fungal infections are more prevalent and often more serious than have been appreciated, and investment is required in basic research and public engagement to address the clinical challenge they impose. Although new antifungals and better diagnostics are under development, the impact of currently available tools and interventions on mortality rates owing to fungal infection has not changed significantly in recent years. Therefore, efforts dedicated to understanding and exploiting our knowledge of the immunology of fungal infections are highly relevant in addressing the global fungal infection problem.

Recent research has defined the immunopathology of many fungal diseases. In some cases, pathology is driven by fungal invasion and virulence, whereas in others, it is mediated by over-activation of the inflammatory response to the fungus. Therefore, it is essential to be able to understand the immunopathology of specific fungal disease settings. Significant strides have been made in understanding how the immune system is activated and suppressed by fungal infections, and new opportunities are presenting themselves to be able to manipulate the immune response using immune agonists or inhibitors of inflammation. Next-generation sequencing has revolutionized our understanding of human genes that predispose patients to specific fungal infections and these have illuminated fundamental mechanisms of immune surveillance and recognition. Increasingly, it has been recognized that the outcome of an infection is mediated not only by the fungus and the immune response, but also by the genetic profile of each patient, and the modulatory influences of the patient's microbiome and mycobiome. Embracing these insights presents new opportunities for the future of vaccine development, adjunct immunotherapy and personalized approaches to protecting and treating patients from fungal infections.
